# Diurnal variations in muscle and liver glycogen differ depending on the timing of exercise

**DOI:** 10.1186/s12576-021-00821-1

**Published:** 2021-11-21

**Authors:** Kaito Iwayama, Yoko Tanabe, Fumiya Tanji, Takahiro Ohnishi, Hideyuki Takahashi

**Affiliations:** 1 Faculty of Budo and Sport Studies, Tenri University, 80 Tainoshocho, Tenri, Nara, 632-0071 Japan; 2 Faculty of Health and Sport Sciences, University of Tsukuba, Ibaraki, Japan; 3 Sport Medical Science Research Institute, Tokai University, Kanagawa, Japan; 4 Medical Center, Japan Institute of Sport Sciences, Tokyo, Japan

**Keywords:** Liver, Muscle, Glycogen, Post-absorptive exercise, Post-prandial exercise

## Abstract

It has been suggested that glycogen functions not only in carbohydrate energy storage, but also as molecular sensors capable of activating lipolysis. This study aimed to compare the variation in liver and muscle glycogen during the day due to different timing of exercise. Nine healthy young men participated in two trials in which they performed a single bout of exercise at 70% of their individual maximal oxygen uptake for 60 min in the post-absorptive (morning) or post-prandial (afternoon) state. Liver and muscles glycogen levels were measured using carbon magnetic resonance spectroscopy (^13^C MRS). Diurnal variations in liver and muscle glycogen compared to baseline levels were significantly different depending on the timing of exercise. The effect of the timing of exercise on glycogen fluctuation is known to be related to a variety of metabolic signals, and the results of this study will be useful for future research on energy metabolism.

## Background

The evidence regarding the health benefits of regular physical activity is well established, and previous research demonstrates that virtually everyone benefits: men and women, young children to older adults, women who are pregnant or postpartum, people living with a chronic disease, or people attempting to reduce their risk of disease [27]. Regular exercise can also be expected to achieve and maintain desired body composition, such as weight loss and weight gain inhibition, due to increased energy expenditure [27]. According to the World Health Organization physical activity guidelines, adults are recommended to undergo 150–300 min of moderate-intensity or 75–150 min of vigorous-intensity physical activity, or an equivalent combination of moderate-intensity and vigorous-intensity aerobic physical activities per week to maintain a healthy body [4]. However, while these guidelines indicate the time, frequency, and intensity of exercise, they do not describe the timing of exercise, such as post-absorptive or post-prandial states.

Recently, the timing of exercise has received much attention due to its influence on various factors, such as food intake [2], circadian rhythm [36, 41], energy substrates [39], body composition [3, 10, 24, 33], the adaptation of training [6, 38], and glucose metabolism [23, 37]. While there is still disagreement regarding some of the above, there is general consensus on the energy substrates, i.e., exercise performed in the post-absorptive state induces higher fat oxidation than exercise performed in the post-prandial state [39]. It has long been known that pre-exercise nutritional status affects energy substrates during exercise [7, 14]. Additionally, post-absorptive exercise increases 24-h fat oxidation, even in energy-balanced conditions [15, 31]. The body condition after an overnight fast is characterized by no energy intake since the supper of the previous night, moreover, overnight fasting is the only way to induce a post-absorptive state in people with traditional eating habits of three meals daily. During overnight fasting, substrate oxidation predominantly shifts from glycogenolysis to fatty acid oxidation [30], and hormonal changes activate several transcription factors and regulate gene expression [11]. A possible physiological background for these aspects is that glycogen variability is involved in whole-body energy metabolism. Glycogen in the liver and muscles is a storage source of energy and its quantity affects whole-body energy metabolism. Specifically, decreased liver glycogen levels stimulate lipolysis in adipose tissue through a central nervous system-mediated mechanism [20], and decreased muscle glycogen levels trigger sequential events, including the dissociation of the AMP-activated protein kinase (AMPK–glycogen interaction, enhanced activity and altered intracellular localization of AMPK; and the upregulated expression of genes associated with fat oxidation, such as carnitine palmitoyltransferase, fatty acid translocase, and hormone-sensitive lipase [26]. In this way, in the liver and muscle glycogen is not only a source of energy, but also a regulator of whole-body energy metabolism. Nevertheless, none of the previous studies has reported fluctuations in liver and muscle glycogen levels due to post-absorptive or post-prandial exercise.

This study aimed to clarify the effect of exercise in the morning or afternoon, i.e., post-absorptive or post-prandial, on liver and muscle glycogen fluctuations during the day. Liver and muscle glycogen levels were measured over two trials, including a 60-min exercise session in the morning before breakfast or in the afternoon using carbon magnetic resonance spectroscopy (^13^C MRS), which is non-invasive.

## Methods

This study entailed a randomized repeated measures design including two trials with exercise sessions performed in the morning or afternoon; the two trials were separated for at least 1 week.

Each trial consisted of two consecutive days. On the first day (day 1) of each experiment, participants were permitted to engage in normal activities, such as walking up and down stairs, until arriving at the laboratory. Thereafter, they were instructed to refrain from performing strenuous exercise and consuming beverages, such as energy, caffeinated, or alcoholic drinks, except for experimental meals. Participants arrived at the laboratory before 17:30. Their body composition was subsequently measured using the bioimpedance method (InBody 770; InBody, Tokyo, Japan). They consumed supper at 18:30 and went to bed at 23:00. On the second day (day 2), two meals (breakfast at 09:00 and lunch at 13:00) were provided, and participants ran at 70% of their VO_2max_ for 60 min, beginning at 07:00 (morning) or 16:00 (afternoon) on a treadmill. Participants were instructed to remain awake and to maintain a sedentary position, except when performing prescribed exercise sessions.

### Participants

Nine healthy young men were recruited for the present study after providing written informed consent. Participants’ mean physical characteristics were as follows: age, 23.4 ± 1.3 years; height, 170.1 ± 1.5 cm; body weight, 59.3 ± 1.0 kg; and body fat, 10.1 ± 0.7%, maximal oxygen uptake (VO_2max_) was 60.7 ± 1.4 ml/kg/min. At the time of the study, no participant had any medical condition or was taking medications or smoking. This study was approved by the Ethics Committee of the Japan Institute of Sports Sciences (IRS-2017-048).

### Pre-study evaluation

To determine the workload corresponding to 70% of the individual VO_2max_, all participants performed a graded exercise test including submaximal and maximal tests using a treadmill (Ohtake Root Kogyo, Japan) at least 1 week before the main experiment. The initial running speed was set at 9.0 km/h and was then increased by 1.2 km/h every 3 min at each subsequent stage up to a lactate concentration exceeding 4.0 mmol/L. Each stage was separated by a 1-min rest, and the rest was extended to 3 min when the lactate concentration exceeded 4.0 mmol/L. Then, the following running speed was set to the stage before the lactate concentration exceeded 4.0 mmol/L and was increased by 0.6 km/h every 1 min until the participants reached voluntary exhaustion. A capillary blood sample was taken from fingertips to analyze the lactate concentration (Lactate Pro 2, Arkray Co., Ltd., Kyoto, Japan) immediately before the test, after each running stage, and after 1, 3, and 5 min of exercise to exhaustion.

Oxygen uptake was considered to be maximal when at least two of the following four criteria were met: (1) VO2 reached a plateau; (2) heart rate exceeded 90% of the age-predicted maximal value; (3) respiratory exchange ratio increased above 1.10, and (4) ratings of perceived exertion at the end of the test ≥ 19. The highest VO_2_ for two consecutive 15 s during the test was taken as the VO_2max_. Respiratory gas was continuously collected using the breath-by-breath method (AE310S, Minato Medical Science Co., Osaka, Japan). Regression analysis revealed that the relative oxygen uptake and treadmill running velocity corresponded to 70% of the individual VO_2max_.

### Experimental meals

Experimental meals were designed to achieve energy-balanced conditions, assuming a resting metabolic rate of 24.0 kcal/kg/day according to estimated energy requirements for Japanese individuals [1]. The physical activity factor was assumed to be 1.75 (2471 ± 39 kcal/day) on day 1 and 1.85 on day 2 (1732 ± 33 kcal for breakfast and lunch) based on a previous study using a room-size metabolic chamber [17]. Participants consumed 6.2 ± 0.1 g/kg of body weight of carbohydrates (370 ± 7 g/day on day 1 and 4.4 ± 0.0 g/kg of body weight (260 ± 5 g for breakfast and lunch on day 2. Experimental meals comprised 15% protein, 25% fat, and 60% carbohydrate; these values were expressed as percentages of the total energy intake.

### Glycogen measurements

Muscle and liver glycogen contents were measured using ^13^C MRS at 20:00 on day 1, and at 06:00, 08:00, 12:00, 15:00, and 17:00 on day 2. Muscle glycogen was assessed using the calf, as it is one of the major muscles that contract during running [5]. ^13^C MRS was performed using a clinical 3-Tesla superconducting magnetic resonance system (Magnetom Verio and Magnetom Skyra, Siemens, Germany) as previously described [18, 35]. Briefly, the ^13^C MRS muscle spectra were collected in 15-min blocks at 200-ms intervals, resulting in 4500 acquisitions using a ^13^C–^1^H double-tuned surface coil, 10 cm in diameter (Takashima Seisakusho, Tokyo, Japan) that was set on the calf muscle. Muscle glycogen levels were quantified by comparing them with an external standard solution (120 mM glycogen from oysters and 50 mM KCl). Similarly, the ^13^C MRS liver spectra were collected in 16-min blocks at 160-ms intervals, resulting in 6000 acquisitions using the same coil as that for muscle measurement; the coil was set on the right side of the trunk by the liver. Liver glycogen levels were quantified by comparing them with an external standard solution (200 mM glycogen from oysters and 150 mM KCl). When the first measurement was taken in both the muscle and liver, the coil position was marked on the skin with a felt-tipped pen to ensure that ^13^C MRS data were collected at the same position during subsequent measurements.

### Blood sampling and analyses

Blood samples were collected from the antecubital vein in commercially available vacuum-sealed serum collection tubes (Nipro, Osaka, Japan) at the same time as glycogen measurements. Serum samples were obtained by centrifugation at 3000 rpm for 10 min at 4 °C and were stored at − 80 °C until analysis. From the obtained samples, insulin, and triglyceride levels were measured in an independent laboratory (LSI Medience Corporation, Tokyo, Japan). Blood glucose levels were determined by blood samples taken from participants’ fingertips (Medisafe FIT, Terumo, Tokyo, Japan) [13].

### Experimental exercise and physical activity

Participants ran for 60 min at 70% of their VO_2max_ on a treadmill (Ohtake Root Kogyo, Japan).

During the experimental 60-min running session, respiratory gas was continuously collected using the breath-by-breath method (AE310S, Minato Medical Science Co., Osaka, Japan). Immediately after completing the 60-min exercise, lactate concentration was measured by blood samples taken from participants’ fingertips.

Non-exercise-related activity was estimated using a hip-worn accelerometer device (ActiGraph GT9X Link, ActiGraph, Pensacola FL), which was validated using a three-axial accelerometer and data filtering technology [28]. Participants wore accelerometers upon wakening on day 1 until the final glycogen measurement on day 2, except during experimental exercise.

### Statistical analyses

Data in the main text and figures are presented as means ± standard errors. To determine the sample size, the effect size (d) from the present data was expected to be 0.7 for the comparison of changes in muscle and liver glycogen fluctuation during the day. The results of the power analysis using G*Power version 3.1.9.2 (University of Dusseldorf, Germany) indicated a minimum sample size of eight to ensure a power of 0.8 and an α level of *P* < 0.05. Considering the possibility of dropouts, we recruited nine participants. Mean condition pair values were compared using a paired *t* test. Muscle and liver glycogen levels, respiratory gas analysis during exercise sessions, and blood parameters between both trials were compared using a two-way repeated-measures analysis of variance to identify the main effect of trial and time. The interaction between trial and time was determined using Bonferroni’s correction with post hoc pairwise comparisons. Changes from baseline in muscle and liver glycogen between both trials were compared using a two-way repeated-measures analysis of variance to identify the main effect of trial and time. The interaction between trial and time was determined using Bonferroni’s (trial) and Dunnett’s (time) correction with post hoc pairwise comparisons. Statistical significance was set at *P* < 0.05. All statistical analyses were performed using SPSS statistical software version 24 (IBM Japan, Tokyo, Japan).

## Results

All participants completed both trials and there were no significant differences in body mass, body fat, or fat-free mass between trials.

During the experimental exercises in the two trials, participants ran on a treadmill at a speed equivalent to 70% of their VO_2max_ for 60 min (12.3 ± 0.4 km/h). This intensity was lower than the lactate threshold of each individual calculated in the pre-study evaluation (14.8 ± 0.6 km/h). No significant differences were observed in the average heart rate (morning, 153 ± 4 bpm; and afternoon, 150 ± 3 bpm; *P* = 0.12), post-exercise blood lactate (morning, 1.7 ± 0.4 mM; and afternoon, 1.5 ± 0.3 mM; *P* = 0.16), and the rating of perceived exertion (morning, 11.3 ± 0.9; and afternoon, 11.1 ± 0.7; *P* = 0.65) between trials. The results of respiratory analysis during exercise are shown Table 1.

**Table 1:** Results of respiratory analyses

	10 min	20 min	30 min	40 min	50 min	60 min	Average
O_2_ uptake (ml/min)							
Morning	2494 ± 51	2623 ± 52	2618 ± 51	2620 ± 54	2614 ± 56	2624 ± 55	2599 ± 51*
Afternoon	2470 ± 45	2577 ± 46	2569 ± 50	2566 ± 54	2564 ± 55	2561 ± 54	2551 ± 49
CO_2_ production (ml/min)							
Morning	2315 ± 70	2439 ± 65	2423 ± 65	2414 ± 72	2414 ± 73	2419 ± 71	2404 ± 68**
Afternoon	2408 ± 66	2541 ± 67	2531 ± 67	2521 ± 73	2508 ± 75	2506 ± 77	2502 ± 69
Ventilation (L/min)							
Morning	66.8 ± 3.2	72.0 ± 3.2	73.6 ± 3.5	74.5 ± 4.0	75.1 ± 4.2	75.4 ± 4.2	72.9 ± 3.6
Afternoon	68.6 ± 3.1	74.6 ± 3.2	75.3 ± 3.2	75.1 ± 3.2	75.3 ± 3.5	75.8 ± 3.5	74.1 ± 3.2
Respiratory exchange ratio							
Morning	0.93 ± 0.02	0.93 ± 0.01	0.92 ± 0.01	0.92 ± 0.01	0.92 ± 0.01	0.92 ± 0.01	0.92 ± 0.01**
Afternoon	0.97 ± 0.01	0.99 ± 0.01	0.98 ± 0.01	0.98 ± 0.01	0.98 ± 0.01	0.98 ± 0.01	0.98 ± 0.01

Values are shown as mean ± standard errors. *P < 0.05, **P < 0.01 vs afternoon trial

**Fig. 1:**
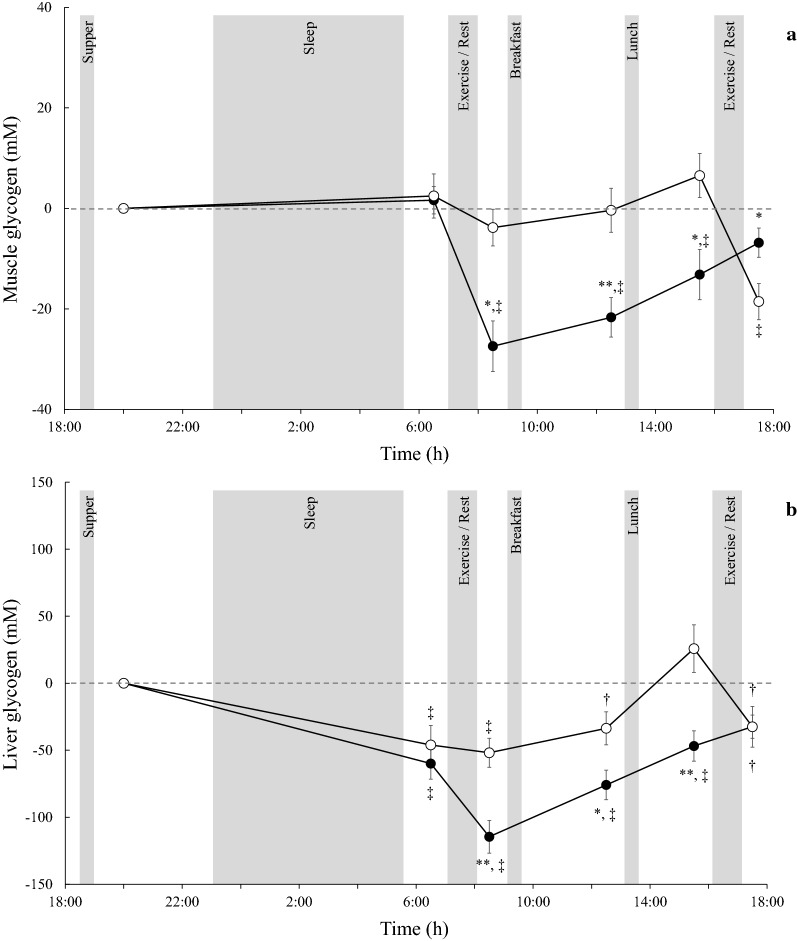
Change from baseline (at 20:00 on day 1) in muscle (**a**) and liver (**b**) glycogen in the morning (closed circles) and afternoon trials (open circles). Values are shown as means ± SEs. *P < 0.05 vs. afternoon trial. **P < 0.01 vs. afternoon trial. †*P* < 0.05 vs. baseline. ‡*P* < 0.05 vs. baseline

Table 2 shows the time course of glycogen levels in the muscle and liver. Significant main effects of trial (*P* < 0.05) and time (*P* < 0.01) and time and trial interactions (*P* < 0.01) were observed for muscle glycogen levels. Significant main effects of time (*P* < 0.01) and trial and time interactions (*P* < 0.01) and a significant trial tendency (*P* = 0.06) were observed when analyzing liver glycogen levels. Figure 1 shows the change from baseline in muscle and liver glycogen. Significant main effects of time (*P* < 0.01) and time and trial interactions (*P* < 0.01) were observed for change from baseline in muscle glycogen, although the main effect of trial (*P* = 0.11) was not statistically significant. Similarly, significant main effects of trial (*P* < 0.05), time (*P* < 0.01) and time and trial interactions (*P* < 0.01) were observed for change from baseline in liver glycogen.

**Table 2:** Results of muscle and liver glycogen measurements

	Day 1	Day 2				
	20:00	6:00	8:00	12:00	15:00	17:00
Muscle glycogen (mM)						
Morning	86 ± 7	88 ± 7	59 ± 7**	65 ± 6**	73 ± 5**	80 ± 6
Afternoon	90 ± 6	93 ± 6	87 ± 6	90 ± 7	97 ± 7	72 ± 7
Liver glycogen (mM)						
Morning	245 ± 20	185 ± 14	130 ± 16**	169 ± 18*	202 ± 18**	213 ± 20
Afternoon	239 ± 15	193 ± 17	187 ± 14	206 ± 14	266 ± 21	207 ± 20

Values are shown as mean ± standard errors. *P < 0.05, **P < 0.01 vs afternoon trial

**Fig. 2:**
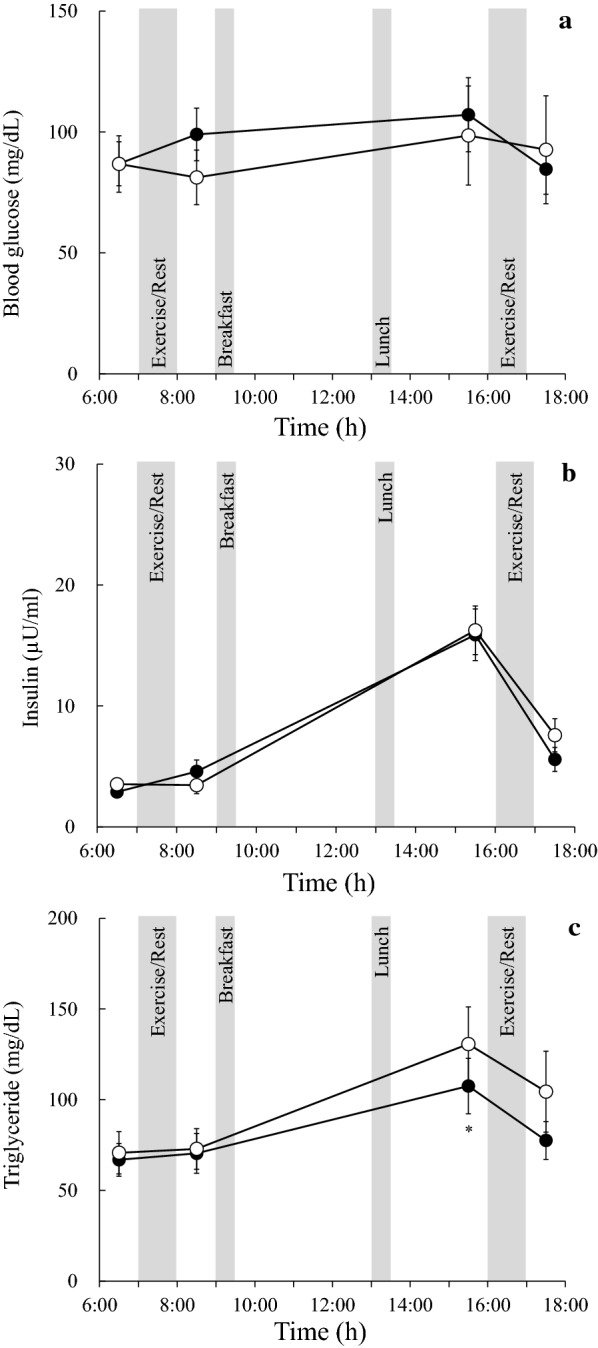
Changes in blood glucose (**a**), insulin (**b**), and triglyceride (**c**) levels in the morning (closed circles) and afternoon trials (open circles). Values are shown as means ± SEs. *P < 0.05 vs. afternoon trial

Significant main effects of trial (*P* < 0.05) and time (*P* < 0.01) were observed for blood glucose levels, although the main effect of trial and time interaction was not statistically significant (Fig. 2a). Significant main effects of time were observed on serum insulin levels (*P* < 0.01), although the main effect of trial and time interaction was not statistically significant (Fig. 2b). Significant main effects of time (*P* < 0.01) and trial and time interactions (*P* < 0.05) were observed on serum triglyceride levels. At 15:30, serum triglyceride levels were significantly higher in the afternoon than in the morning trial (*P* < 0.05, Fig. 2c).

Participants maintained an inactive and sedentary lifestyle during both experimental days, except when they conducted exercises. No significant differences were observed between the morning and afternoon trials in non-exercise physical activity (day 1, 325 ± 27, 317 ± 24 counts/min, *P* = 0.81; and day 2; 139 ± 9, 138 ± 5 counts/min, *P* = 0.86) and step counts (day 1, 6746 ± 735, 6687 ± 651, *P* = 0.94; day 2, 1733 ± 239, 1639 ± 176, *P* = 0.28).

## Discussion

The purpose of this study was to determine the effect of the timing of exercise on fluctuations in liver and muscle glycogen levels. Our main findings were that exercise in the morning was associated with relatively lower liver and muscle glycogen during the day compared to exercise in the afternoon.

Compared with baseline values (i.e., at 20:00 on day 1), liver glycogen levels decreased after overnight fasting in the morning (-23%) and afternoon (-21%) trials. A key function of liver glycogen is to maintain blood glucose levels by releasing glucose into the bloodstream [12]; therefore, its degradation after overnight fasting is reasonable [18]. The morning trial induced further low liver glycogen levels (-46%, at 08:00 on Day 2) by experimental exercise, and liver glycogen remained significantly decreased relative to baseline, even at the last measurement. However, there was no significant difference in liver glycogen levels before experimental exercise compared to baseline in the afternoon trial. This was due to the fact the participants consumed enough carbohydrates at breakfast and lunch to replenish the glycogen degradation by the overnight fast. Consequently, it was found that liver glycogen fluctuations during the day differ depending on exercise timing. A shortage in liver glycogen directly facilitates lipolysis in white adipose tissue by activating a liver–brain–adipose neurocircuitry, this demonstrates the presence of a “glycogen depletion signal” [20, 40]. The fat utilization signal may continue to increase fat oxidation after exercise in the morning as decreased liver glycogen levels remain severely depressed after breakfast [15]. In addition to fat metabolism, plasma glucose clearance rates are higher in the morning compared with the evening in response to similar glucose intake [34]. Since increased liver glycogen synthesis leads to improved glucose tolerance [29], a significant transient decrease in liver glycogen by exercise may be beneficial in improving glucose tolerance. It has been suggested that optimizing the timing of exercise is beneficial for improving glucose homeostasis [9] and diurnal variation in liver glycogen with different timing of exercise would provide useful insights for carbohydrate/fat metabolism.

Muscle glycogen levels did not decrease after overnight fasting in the morning (+ 2%) and afternoon (+ 3%) trials compared with each baseline value. Diurnal muscle glycogen variation has been observed to be markedly small on sedentary days [18, 35] because muscle glycogen does not contribute to the maintenance of blood glucose. Thus, there were no significant differences in pre-exercise muscle glycogen levels compared to baseline levels in each study, but the diurnal variation differed significantly depending on the timing of exercise. The respiratory exchange ratio during the experimental exercise was significantly lower in the morning trial than in the afternoon trial (Table 1). Nonetheless, the net change in muscle glycogen due to experimental exercise did not differ between trials. Previous research indicates that net muscle glycogen breakdown is similar between post-absorptive and post-prandial exercise, but there is a significant increase fat oxidation and decrease in the intramyocellular triglyceride in type I muscle after post-absorptive compared to post-prandial exercise [8]. It has been suggested that intramyocellular triglyceride content is a marker or mediator of muscle insulin resistance [22]. Furthermore, muscle glycogen is not only a carbohydrate energy reserve, but also a molecular sensor capable of activating signaling pathways in response to exercise, including the nuclear translocation of AMPK and upregulation of genes responsible for fat oxidation [26]. Additionally, exercise-induced muscle glycogen depletion can increase subsequent insulin sensitivity and prevent glucose from being diverted to de novo lipogenesis [21]. When considering carbohydrate/fat metabolism and glucose tolerance, it is potentially beneficial to know the timing of exercise and muscle glycogen fluctuations.

In summary, the diurnal fluctuations in liver and muscles glycogen varied depending on the timing of exercise. Previous studies suggest that the timing of exercise may affect energy metabolism over 24 h by altering the pattern of glycogen fluctuations [15, 16, 31]. Furthermore, acute [8, 32] and chronic [24, 38] post-absorptive exercise have been observed to induce different metabolic adaptations than post-prandial exercise, and have been associated with diurnal variation of glycogen storage. Future studies are needed to evaluate the effect of the timing of exercise on glycogen fluctuations in the liver and muscles over a longer period.

One limitation of the present study is that it did not measure liver and muscle glycogen levels after exercise in the afternoon trial, that is, the effect of supper and sleep on post-exercise glycogen fluctuation. Thus, the effect of morning or afternoon exercise on the circadian rhythm of glycogen variability remains unclear. Although the nocturnal energy expenditure and substrate did not differ depending on the timing of exercise [15, 17], it would be interesting to investigate liver and muscle glycogen levels the following morning after supper and sleep to examine the relationship between glycogen fluctuations and linked physiological indicators. Another limitation is that the post-exercise carbohydrate intake timing might not have been optimal for glycogen resynthesis. Consuming carbohydrates immediately after exercise is better for glycogen resynthesis than consuming carbohydrates 2 h after exercise [19]. For the purpose of glycogen measurement, participants in this study consumed breakfast 90 min after exercise rather than immediately in the morning trial. However, differences in intake timing do not affect glycogen synthesis when evaluated over 8 h or 24 h [25]. Therefore, we believe that the effect of dietary intake timing in this study on eventual glycogen storage is negligible. Finally, the intensity and duration of the experimental exercise may be too intense for the general population. The energy expenditure during exercise, which is feasible for many people, may be lower than in the experimental exercise of the present study, and consequently, the variability in glycogen may be smaller.

## Conclusions

Exercise performed in the morning before breakfast was associated with a relative decrease in liver and muscle glycogen during the day compared to exercise performed in the afternoon. The effect of the timing of exercise on glycogen fluctuation, which has been shown to be related to a variety of metabolic signals, will be useful in future studies of energy metabolism. The results of this study may also be applied to consider the optimal exercise timing for patients with diabetes.

## Data Availability

The datasets used and/or analyzed during the current study are available from the corresponding author on reasonable request.
